# Whole gene expression profile in blood reveals multiple pathways deregulation in R6/2 mouse model

**DOI:** 10.1186/2050-7771-1-28

**Published:** 2013-10-23

**Authors:** Daniela Diamanti, Elisa Mori, Danny Incarnato, Federico Malusa, Costanza Fondelli, Letizia Magnoni, Giuseppe Pollio

**Affiliations:** 1 Siena Biotech S.p.A., strada del Petriccio e Belriguardo, Siena 35, 53100, Italy; 2 HuGeF (Human Genetics Foundation), via Nizza 52, Torino, Italy

**Keywords:** Huntington disease, R6/2, Gene expression, Blood

## Abstract

**Background:**

Huntington Disease (HD) is a progressive neurological disorder, with pathological manifestations in brain areas and in periphery caused by the ubiquitous expression of mutant Huntingtin protein. Transcriptional dysregulation is considered a key molecular mechanism responsible of HD pathogenesis but, although numerous studies investigated mRNA alterations in HD, so far none evaluated a whole gene expression profile in blood of R6/2 mouse model.

**Findings:**

To discover novel pathogenic mechanisms and potential peripheral biomarkers useful to monitor disease progression or drug efficacy, a microarray study was performed in blood of R6/2 at manifest stage and wild type littermate mice. This approach allowed to propose new peripheral molecular processes involved in HD and to suggest different panels of candidate biomarkers. Among the discovered deregulated processes, we focused on specific ones: complement and coagulation cascades, PPAR signaling, cardiac muscle contraction, and dilated cardiomyopathy pathways. Selected genes derived from these pathways were additionally investigated in other accessible tissues to validate these matrices as source of biomarkers, and in brain, to link central and peripheral disease manifestations.

**Conclusions:**

Our findings validated the skeletal muscle as suitable source to investigate peripheral transcriptional alterations in HD and supported the hypothesis that immunological alteration may contribute to neurological degeneration. Moreover, the identification of altered signaling in mouse blood enforce R6/2 transgenic mouse as a powerful HD model while suggesting novel disease biomarkers for pre-clinical investigation.

## Background

Huntington disease (HD) is an autosomal dominant neurodegenerative disorder characterized by progressive atrophy of specific brain areas with consequent alterations of motor and cognitive functions, including psychiatric disturbances, weight loss, as well as metabolic, neuroendocrine and immunological alterations [1-3]. The cause of this fatal disease is an aberrant expansion of CAG trinucleotide in the exon 1 of *HTT* gene, translating into a polyglutamine tract (polyQ) at the N-terminus, and conferring gain-of-function and loss-of-function to wild type huntingtin protein. In healthy individuals, CAG number is included in the range of 6 to 26, while HD patients have more than 36 Gln stretch [1] with an inverse relationship between polyQ length and the age of onset. The aberrant polyQ tract results in Huntingtin protein misfolding, which generates insoluble intracellular inclusions and aggregates, important hallmarks of the disease.

Despite extraordinary efforts in understanding the HD pathogenesis, the exact molecular mechanisms responsible for this devastating disorder are still unknown and probably multiple parallel processes may contribute, representing potential therapeutic targets [4,5]. Striking evidences support an important role of transcriptional abnormalities in HD pathogenesis [6]. Mutant Huntingtin protein (mut-HTT) and its aggregates could modulate gene transcription by entering the nucleus and altering the transcriptional machinery either directly, through DNA binding [7], or sequestering important transcription factors [8].

Even though no animal models currently in use encompass all disease features, transgenic mice carrying either full length or mut-HTT fragments represent an invaluable instrument to study pathological mechanisms and to test efficacy and toxicity of small molecules in pre-clinical settings [9-11]. Numerous behavioral and neurological symptoms similar to what seen in HD patients have been also observed in transgenic mouse models [11,12] including those expressing only short fragments of mut-HTT, as R6/2 mice [13]. R6/2 is one of the first HD transgenic mouse model created, expressing only the N-terminal fragment of HTT (exon 1) and it is characterized by short survival and development of pathological features mimicking human stages of disease [13,14]. For this reason, transcriptional and behavioral alterations have been intensely studied in R6/2, which has being commonly employed not only in pre-clinical drug testing but also in peripheral investigations, often realized in parallel with HD patients [15,16]. Stemming from those observations, although HD is mainly a CNS disorder, ubiquitous expression of both normal and mut-HTT in the whole body [17] has moved the attention toward peripheral dysfunctions, increasing the knowledge on HD etiology and other disease manifestations, and aiding the finding of new biomarkers [18,19], as evidenced by the flourishing number of transcriptional and proteomic studies conducted in HD blood [16,20-24].

In this study, we investigated transcriptional dysregulations occurring in R6/2 and wild type littermate mouse blood to reinforce previous observations derived from human studies and to discover novel pathological processes and candidate biomarkers, useful in pre-clinical investigations and in designing therapeutic trials. The transcriptional alterations detected in blood were also analyzed in brain to identify a possible link with central pathological mechanisms, and in other peripheral tissues, like skeletal muscle and skin, to validate them as additional sources of potential biomarkers.

## Findings

### Primary analysis of microarrays

**Figure 1:**
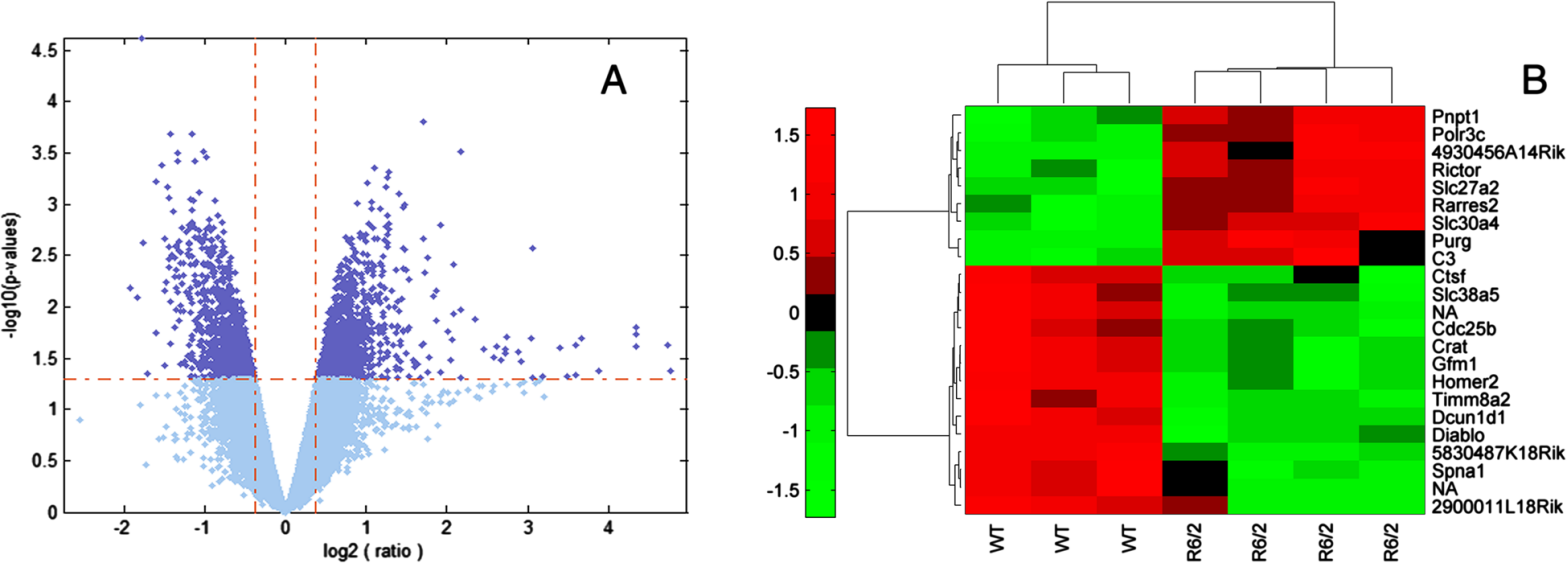
**Microarray results.** Figure shows results of microarray analysis performed on four R6/2 versus three WT mice using LIMMA package: **(A)** Volcano plot shows the –log10 of p-values against the genes biological effect expressed as log2 transformed fold change. Cutoff values are set to p < 0.05 and abs FC > 1.3. **(B)** Bi-dimensional cluster (obtained by using correlation and Euclidean distances between genes and strains, respectively, and applying average linkage method) shows the 23 most significant differentially expressed probe sets [p < 0.001 and abs FC > 2].

To discover differential peripheral gene expression between R6/2 and WT littermate mice, blood samples were collected at 16 weeks of age, when mutant mice were starting to show neurological signs of decline, like abnormal motor behavior and body weight loss, all characteristics of symptomatic state of the HD pathology. Transcriptional investigation was carried through a microarray study performed on individual blood sample of four R6/2 and three WT mice. Microarray analysis showed approximately 2200 transcripts significantly modulated between R6/2 and WT mice, according to selection criteria of p-value (p) lower than 0.05 and absolute fold change (abs FC) higher than 1.3 (Figure 1A). Among these, 1072 probe sets were up-regulated and 1164 were down-regulated. A limited number of transcripts (133) resulted significantly altered in blood with a p < 0.05 and high FC (abs FC > 2). A selection of these most differentially expressed (p < 0.001; abs FC > 2) genes are reported in Figure 1B.

### GSEA analysis of differentially expressed genes

In order to highlight relevant processes that could be altered in blood, we applied Gene Set Enrichment Analysis (GSEA) method on the same dataset. This computational method identifies groups of genes with modest but coordinate changes in the expression between phenotypes by utilizing all transcripts of a microarray dataset and discovering significant differences in terms of pathways or gene sets associated with a particular biological function. Microarray analysis was performed using KEGG, BioCarta and Reactome pathway collections of MSigDB and was enforced by Biological Processes of GeneOntology.

Analyzing KEGG, 92 gene sets resulted up-regulated and 77 down-regulated in mutant mice with respect to wild type mice, and among them, 19 pathways were significantly enriched in R6/2, while 2 were significantly enriched in wild type, having FDR < 25% (Table 1). Reactome database, which contains a wider number of gene sets with respect to KEGG, showed a total number of 309 up-regulated collections, 14 of them with FDR < 25%, and 169 down-regulated collections, but none with a significant modulation (Table 1). By using BioCarta significant modulations were revealed only in 3 up-regulated gene sets among 79 positively regulated and 57 negatively regulated (Table 1).

**Table 1:** GSEA canonical pathways results on three different database (KEGG, Reactome and BioCarta)

	**Gene set name**	**SIZE**	**ES**	**NES**	**NOM p-val**	**FDR q-val**	**FWER p-val**
KEGG	COMPLEMENT AND COAGULATION CASCADES	61	0.69	2.56	0.000	0.000	0.000
	PPAR SIGNALING PATHWAY	60	0.56	2.02	0.000	0.004	0.006
	GLYCINE SERINE AND THREONINE METABOLISM	29	0.60	1.87	0.002	0.023	0.057
	PRIMARY BILE ACID BIOSYNTHESIS	15	0.69	1.77	0.006	0.044	0.137
	RIBOSOME	82	0.45	1.73	0.000	0.052	0.199
	LYSINE DEGRADATION	43	0.48	1.63	0.007	0.126	0.483
	HISTIDINE METABOLISM	28	0.53	1.62	0.020	0.110	0.488
	SYSTEMIC LUPUS ERYTHEMATOSUS	54	0.46	1.62	0.007	0.101	0.505
	PEROXISOME	73	0.43	1.60	0.002	0.105	0.565
	PENTOSE PHOSPHATE PATHWAY	23	0.55	1.59	0.013	0.100	0.582
	BUTANOATE METABOLISM	30	0.51	1.59	0.007	0.095	0.598
	CYSTEINE AND METHIONINE METABOLISM	33	0.47	1.54	0.026	0.131	0.736
	BIOSYNTHESIS OF UNSATURATED FATTY ACIDS	18	0.55	1.53	0.035	0.134	0.770
	TYROSINE METABOLISM	35	0.46	1.51	0.031	0.144	0.822
	PRION DISEASES	32	0.47	1.51	0.024	0.140	0.831
	ONE CARBON POOL BY FOLATE	16	0.55	1.47	0.043	0.177	0.910
	SELENOAMINO ACID METABOLISM	25	0.48	1.46	0.047	0.171	0.916
	FATTY ACID METABOLISM	33	0.45	1.44	0.047	0.193	0.948
	AMINOACYL TRNA BIOSYNTHESIS	41	0.42	1.40	0.048	0.241	0.977
	CARDIAC MUSCLE CONTRACTION	69	−0.5	−1.83	0.000	0.044	0.051
	DILATED CARDIOMYOPATHY	87	−0.46	−1.74	0.000	0.075	0.163
REACTOME	FORMATION OF FIBRIN CLOT CLOTTING CASCADE	31	0.7	2.20	0.000	0.002	0.001
	BILE ACID AND BILE SALT METABOLISM	23	0.73	2.18	0.000	0.001	0.001
	COMPLEMENT CASCADE	23	0.71	2.10	0.000	0.003	0.007
	LIPOPROTEIN METABOLISM	26	0.67	2.05	0.000	0.007	0.019
	RESPONSE TO ELEVATED PLATELET CYTOSOLIC CA2	74	0.53	2.01	0.000	0.010	0.034
	CHYLOMICRON MEDIATED LIPID TRANSPORT	16	0.73	2.00	0.000	0.009	0.039
	INTRINSIC PATHWAY	17	0.73	1.99	0.000	0.008	0.040
	METABOLISM OF AMINO ACIDS AND DERIVATIVES	188	0.45	1.98	0.000	0.009	0.052
	LIPID DIGESTION MOBILIZATION AND TRANSPORT	41	0.58	1.95	0.002	0.011	0.070
	SYNTHESIS OF BILE ACIDS AND BILE SALTS	18	0.69	1.91	0.004	0.016	0.109
	SULFUR AMINO ACID METABOLISM	24	0.61	1.84	0.002	0.035	0.239
	PEPTIDE CHAIN ELONGATION	81	0.45	1.74	0.000	0.094	0.548
	CLEAVAGE OF GROWING TRANSCRIPT IN THE TERMINATION REGION	31	0.51	1.63	0.011	0.238	0.888
	TRANSCRIPTIONAL ACTIVITY OF SMAD2 SMAD3 SMAD4 HETEROTRIMER	36	0.49	1.62	0.007	0.248	0.917
BIOCARTA	COMP PATHWAY	16	0.75	2.02	0.000	0.021	0.017
	INTRINSIC PATHWAY	23	0.67	2.00	0.000	0.012	0.019
	TOB1 PATHWAY	19	0.61	1.70	0.011	0.224	0.412

Table shows statistically up-regulated and down-regulated pathways in R6/2 versus WT mice, and for everyone, number of genes (size) identified in the microarrays, enrichment score (ES), normalized enrichment score (NES), nominal p-value (NOM p-val), false discovery rate (FDR q-val), familywise-error rate (FWER p-val).

Similar findings were obtained by analyzing entire microarray expression dataset in terms of biological processes of GeneOntology collections. In particular, 20 of these differentially modulated processes were linked to metabolism (Additional file 1: Table S1). Results confirmed alteration of metabolic processes in mutant mice as well as coagulation impairment and amino acid, lipid and fatty acid metabolism dysfunctions. Moreover, regulation of muscle and heart contraction processes emerged among enriched processes in WT mice.

To understand information derived from GSEA, we analyzed in details pathways markedly modulated in KEGG database, focusing on the two top enriched collections in R6/2, namely “complement and coagulation cascade” and “PPAR signaling”, together with the two top enriched collections in WT mice, that are “cardiac muscle contraction” and “dilated cardiomyopathy”, emphasizing existing relationships with HD pathology.

#### Complement and coagulation cascade

**Figure 2:**
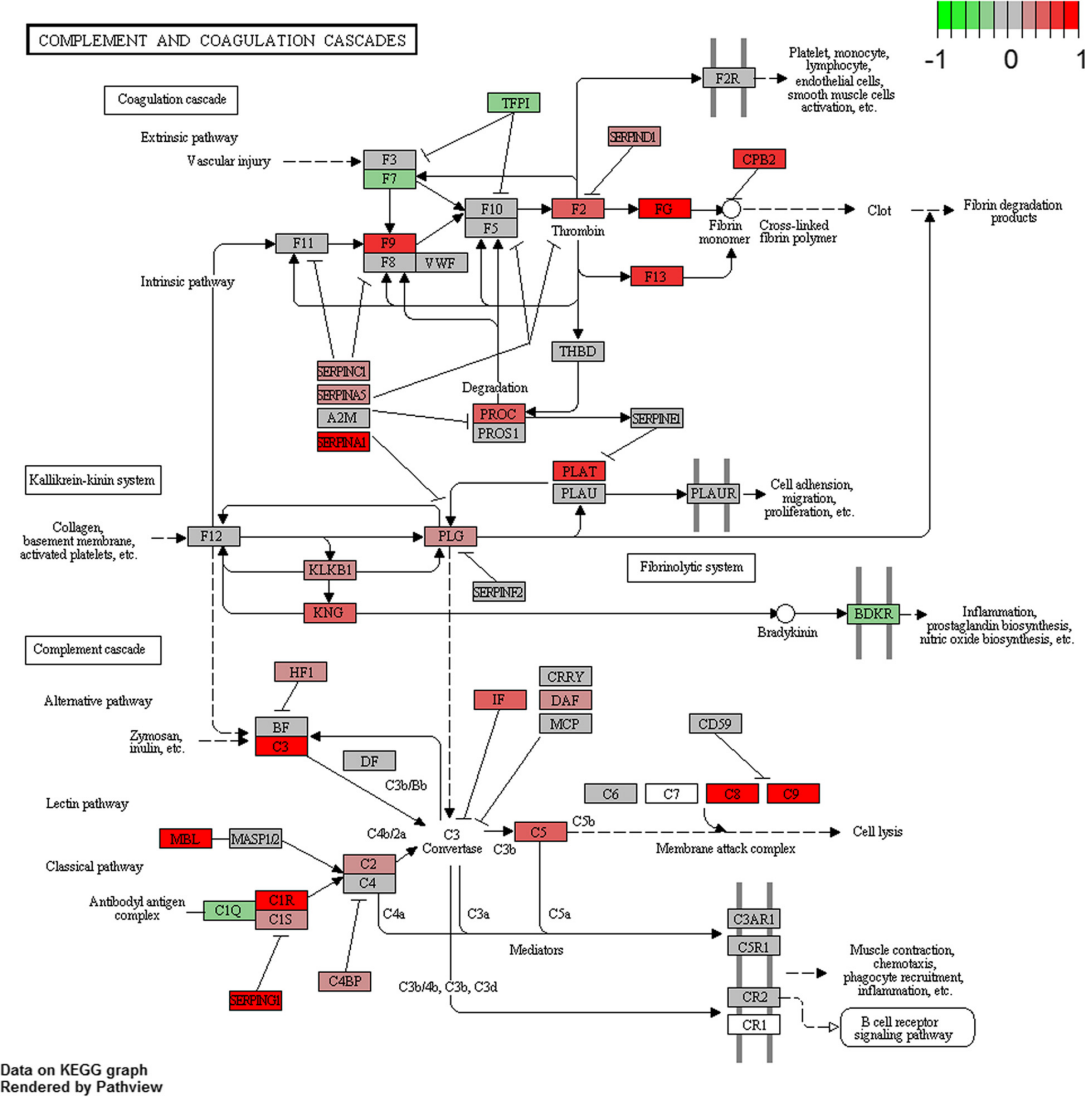
**Complement and coagulation cascades pathway from KEGG.** Genes are colored according to their GSEA calculated scores in R6/2 versus WT analysis (signal-to-noise): the most marked red genes belong to the core enrichment.

Complement and coagulation cascade pathway resulted altered in R6/2 mice in the three database employed with GSEA. Pathway core enrichment obtained from our analysis comprised numerous components of complement system and serpins, as well as many coagulation factors, that resulted up-regulated in blood of R6/2 mice with respect to WT (Figure 2; detailed core enrichment in Additional file 2: Table S2).

The complement system is involved in the first line of host defense against pathogens [25]. Consisting of soluble and membrane embedded proteins, it is a component of the innate immune response, that acts in peripheral tissues and in the brain. Although a protective role linked to clearance of cellular debris has been assigned to several components of the cascade, a relevant effect of its activation in brain is that it may contribute to neuro-inflammation and neuronal loss in neurodegenerative disorders as HD and Alzheimer’s disease [26]. Indeed, an increased number of works debate on the immune system activation in HD [2,27]. Most of them are focused on the complement cascade impairment by accurate investigations at transcriptional and proteomic level, both in blood and brain, to discover potential biomarkers or causative factors of the pathology, respectively [19,23,28]. Here, we discovered for the first time transcriptional alteration of numerous components of the complement system in whole blood of an HD mouse model at manifest stage. Although previous transcriptional studies conducted in HD patients blood [20,21] did not report abnormalities of this pathway, our findings agree with a multi-approaches proteomic investigation performed in HD human plasma samples [23]. In Dalrymple et al. [23], several proteins including some complement components, as C9, were identified as possible disease progression biomarkers, confirming the involvement of acute-phase response and consequent activation of complement cascade in HD patients. In our investigation, no significant alteration of interleukins and cytokines mRNAs was observed in R6/2 blood at this stage, differently from what has been measured in plasma of HD patients and serum of HD animal models [19,29].

The identification of complement and coagulation cascade signaling in murine blood may enforce R6/2 transgenic mouse as HD model to discover pharmacodynamic and disease peripheral biomarkers useful in pre-clinical investigation. Moreover, recent evidences focusing on druggability of pathway components [30,31], evaluated complement factors as potential targets for the treatment of autoimmune and inflammatory diseases, including neurodegenerative disorders.

#### PPAR signaling

Considering KEGG database results, PPAR signaling was the second best enriched pathway in mutant mice. This is intrinsically linked to other downstream processes emerging in the KEGG list of R6/2 up-regulated pathways, such as primary bile acid biosynthesis, biosynthesis of unsaturated fatty acids and fatty acid metabolism, or other pathways related to amino acid metabolism or synthesis (Table 1). Additionally, most of them resulted modulated in the analysis performed by Reactome database (Table 1). A similar metabolic profile was also described in serum of HD patients and in an HD murine model [24].

**Figure 3:**
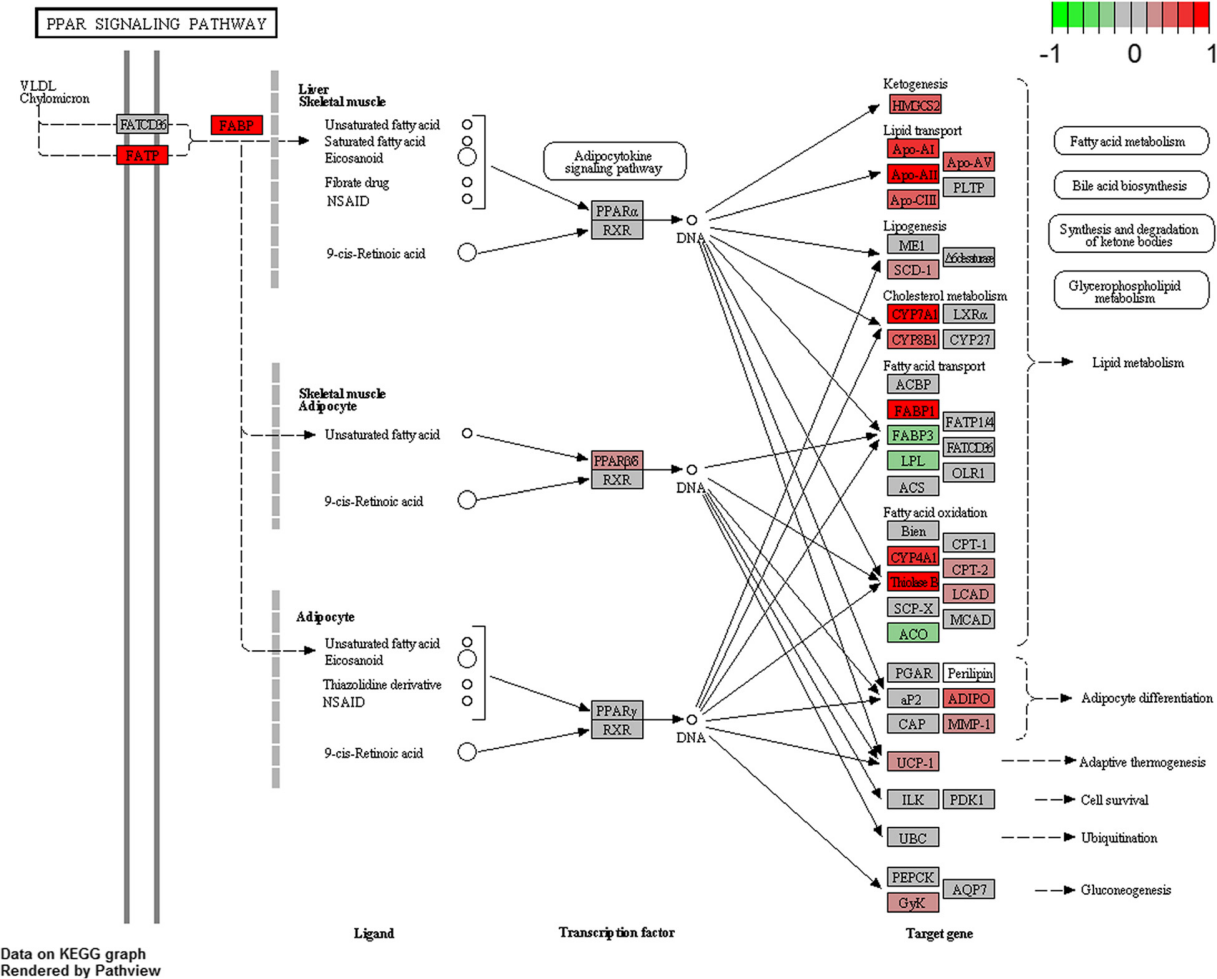
**PPAR signaling pathway from KEGG.** Genes are colored according to their GSEA calculated scores: the most marked red genes belong to the core enrichment.

Peroxisome proliferator-activated receptors, PPARs, belong to the nuclear hormone receptor family, comprising PPARa, PPARg, PPARb/d, and are localized in different tissues where they influence distinct processes. PPARs are ligand-depend transcription factors that form heterodimeric complexes with retinoid-X-receptor RXR, and regulate the expression of target genes involved in carbohydrate, amino acid and lipid metabolism. Figure 3 shows target genes altered in R6/2 blood and belonging to PPAR signaling core enrichment and cognate down-stream processes (detailed core enrichment in Additional file 3: Table S3). The impairment of this signaling has been already seen in different tissues of HD patients and models [32]. It can be noted that many of the genes that contribute to enrich this collection are apolipoproteins, fatty acid binding proteins, *CYP* genes as well as the same *PPARs* and *RXR*. In particular, GSEA revealed adipose tissue dysfunction and adipocitokine signaling alteration that have been already detected in R6/2 mice, where reduced adipokine levels and PPARg target genes linked to adipocyte function and differentiation were observed [33].

The other metabolic processes that emerged as altered in R6/2 blood are well-known for being deregulated in HD. Indeed, we found lipid-linked signaling like fatty acids, bile acids and cholesterol metabolism. Altered cholesterol pathway and its biosynthesis were deeply investigated by Cattaneo and collaborators [34] in patients and animal models, both in peripheral tissues and in CNS [35], demonstrating that cholesterol might have a pivotal role in HD pathogenesis. Moreover, cholesterol dysfunction is closely associated with lipid, triglyceride and fatty acid metabolism that are also impaired in HD [36]. In conclusion, evidences of PPAR signaling impairment in R6/2 blood, mirroring numerous HD systemic metabolic abnormalities [3], may contribute to the identification of new pathogenic mechanisms. Drugs against lipid dysregulation have been already tested in HD and they should be supported by disease biomarkers as PPAR target genes, to monitor therapeutic efficacy.

#### Cardiac muscle contraction and dilated cardiomyopathy

**Figure 4:**
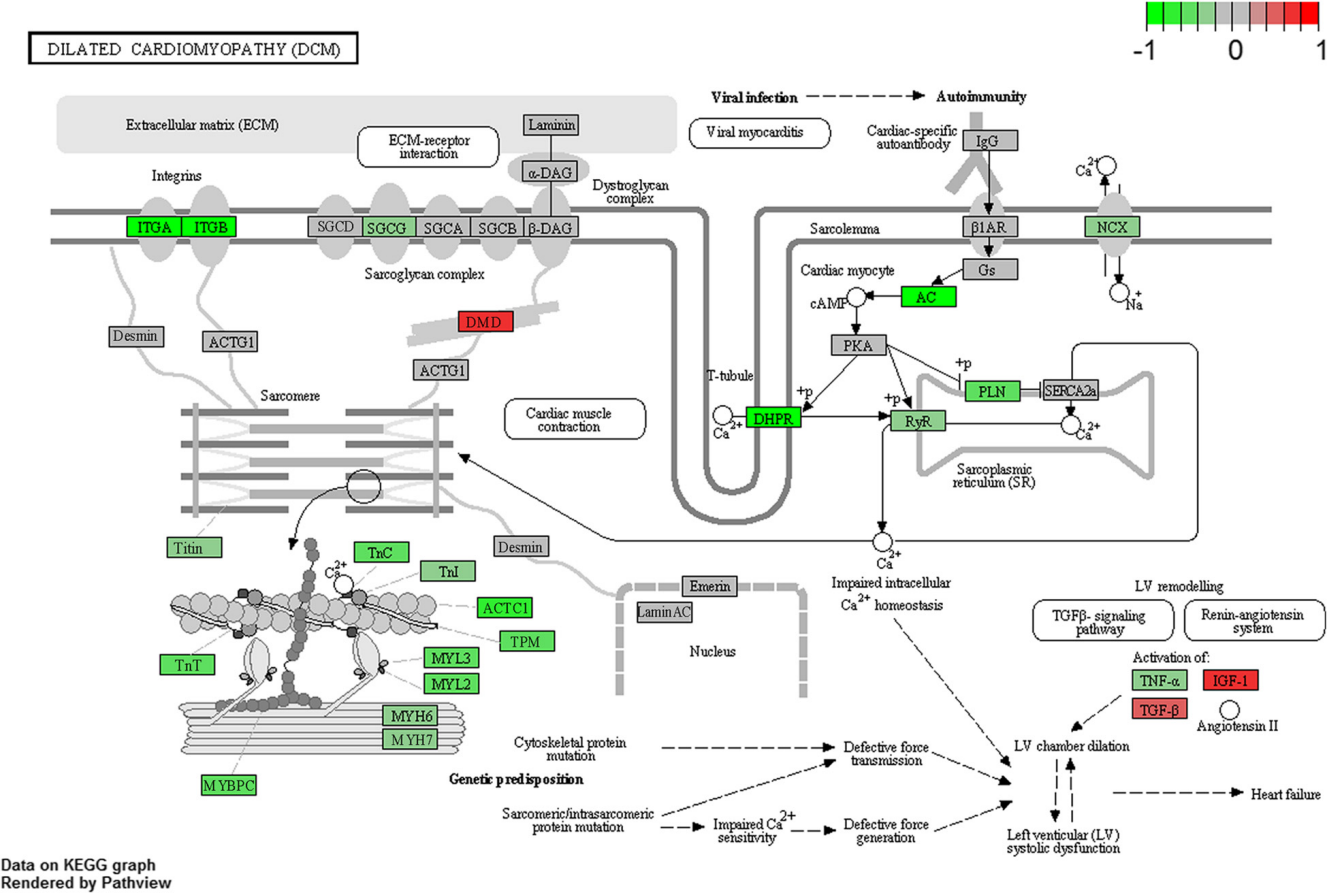
**Dilated cardiomyopathy pathway from KEGG.** Genes are colored according to their GSEA calculated scores.

**Figure 5:**
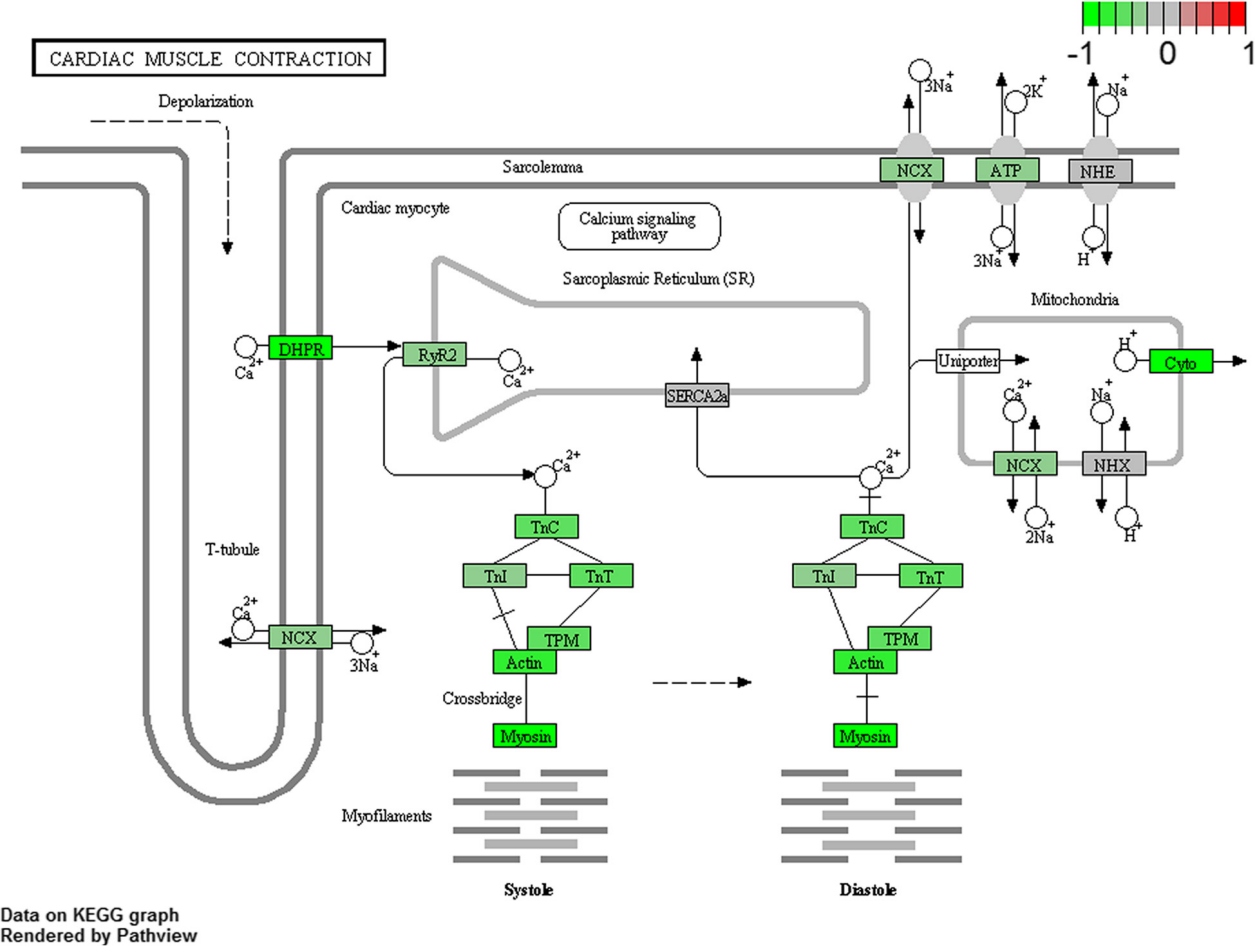
**Cardiac muscle contraction pathway from KEGG.** Genes are colored according to their GSEA calculated scores.

Among all 77 gene set collections enriched in wild type phenotype within KEGG database, only two resulted significantly modulated: dilated cardiomyopathy and cardiac muscle contraction pathways (Figure 4 and 5). Interestingly, other similar pathways (hypertrophic cardiomyopathy and arrhythmogenic right ventricular cardiomyopathy) appeared in the list of modulated collections but with FDR > 25%, p < 0.1. In resulting GSEA core enrichment of cardiac muscle contraction (Figure 5; Additional file 4: Table S4), we found genes linked to calcium efflux and voltage (*Slc8a1*, *Cacna1f*, *Cacng1*, *Cacng3*, *Cacna1s* and *Cacng6*), or expressed in mitochondria and involved in oxidation-reduction processes (*Cox6b2*, *Cox7b2*, *Cox6a2* and *Cox7a1*) as well as genes contributing to the structural integrity of muscle fibers (*Myh7*, *Myh6*, *My13* and *My12*). Moreover, in dilated cardiomyopathy pathway core enrichment (Figure 4; Additional file 5: Table S5), integrins (*Itga1*, *Itga10*, *Itga8* and *Itgb6*) and cellular components of troponin system (*Tnni3*, *Tnnc1* and *Tnnt2*) appeared modulated, besides *Cox* and *Myh* genes.

The two processes found down-regulated in R6/2 blood are related to heart contraction performance, which is monitored by cardiac myocytes excitation with the maintenance of cellular homeostasis through Na^+^ and Ca^2+^ fluxes. Ca^2+^ homeostasis imbalance is considered a relevant cause of many neurodegenerative disorders, including HD [37,38], where impairment of Ca^2+^ physiological equilibrium is generally followed by progressive heart failure and sudden cardiac death. Additionally, Ca^2+^ handling defects may exacerbate mitochondrial dysfunctions [39,40], considered being a relevant mechanism in HD pathogenesis [41], and hence contributing to cardiac failure too [42].

A fascinating remark is that, even if our data were generated from a whole genome expression analysis performed in blood, modulation of these two pathways suggests evidences of cardiac dysfunction in R6/2 mice. Nevertheless, although Huntington’s disease is a neurological disorder, cardiac failure is an important cause of death in a high percentage of HD patients (over 20%) [43]. In addition, a recent work showed direct evidences of cardiac dysfunction in R6/2 [44], highlighting an *in-vivo* impairment of numerous cardiac functions, including myocardial contractility. Our finding agree with what has been previously demonstrated by Mihm et al. [45], about cardiotoxic effects coming from peripheral mut-HTT expression. This study displayed that mut-HTT expression in R6/2 cardiomyocytes could result in severe cardiac systolic and diastolic impairment, showing parallel changes between cardiac and neurological symptoms, and suggesting the use of cardiovascular performance indicators as “state biomarkers” of disease progression. Here, we identified for the first time in HD blood a set of deregulated genes involved in cardiac patho-physiology that should be useful to monitor a drug therapy addressing this HD symptom. Peripheral blood gene expression profiling has been already applied to assess some cardiovascular diseases, as myocardial infarction [46] relying on the ability of blood to operate as a sensor able to capture physiological and pathological modifications, transforming them in gene expression changes [47].

### RT-qPCR

To validate microarray results derived from GSEA, we tested the expressions of selected genes in blood samples through RT-qPCR. For this purpose, mouse blood samples were pooled as indicated in methods section. This step didn’t prejudice the final result as demonstrated by Peng et al. [48]. We analyzed two genes for each mentioned pathway, that were also included in the group of transcripts significantly modulated between R6/2 and WT mice in microarrays (abs FC > 1.3 and p < 0.05). The selected genes consisted of *C3* and *Serping1*, employed to test immunological hypothesis, *Fabp1* together with *Slc27a2* to confirm peripheral involvement of PPAR signaling, and *Tmp2* and *Slc8a1* for validation of cardiac dysfunction (Table 2). This selection was limited to genes with expression levels detectable by RT-qPCR.

**Table 2:** Selected genes investigated by RT-qPCR

**Gene symbol**	**ENTREZ ID**	**GENE NAME**	**Affy Id**	**R6/2 vs WT FC**	**R6/2 vs WT p**
Tpm2	22004	tropomyosin 2, beta	1425028_a_at	-1.8	0.016
Slc8a1	20541	solute carrier family 8 (sodium/calcium exchanger), member 1	1437675_at	-1.36	0.047
C3	12266	complement component 3	1423954_at	4.51	0.000
Serping1	12258	serine (or cysteine) peptidase inhibitor, clade G, member 1	1416625_at	2.6	0.009
Slc27a2	26458	solute carrier family 27 (fatty acid transporter), member 2	1416316_at	3.27	0.000
Fabp1	14080	fatty acid binding protein 1, liver	1448764_a_at	20.35	0.018

Table reports name, symbol and entrez ID (obtained from NCBI database) of each gene. Corresponding Affy id together with relative Fold Change (FC) and p-value (p) obtained by microarray statistical analysis (Section Methods) are also showed.

**Figure 6:**
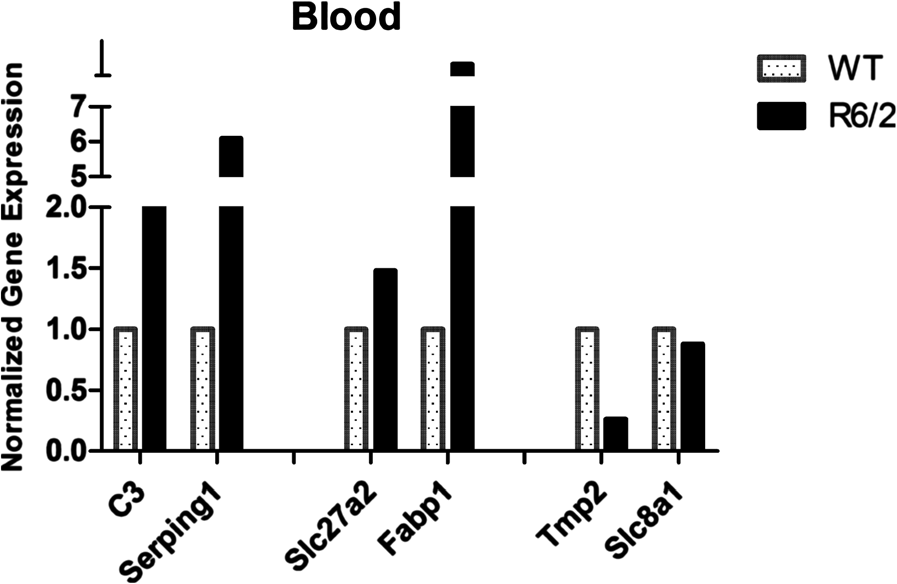
**RT-qPCR in blood.** RT-qPCRs were performed in pooled blood samples, so statistical test was not applied. Normalized expression levels of selected genes confirmed, in blood, the modulation previously observed in microarray, reported in Tab. 3. Since *Fabp1* was measurable in R6/2 but not in WT, this last value was replaced with detection limit of the qPCR machine: the levels in R6/2 resulted in strong up-regulation with respect to WT.

**Figure 7:**
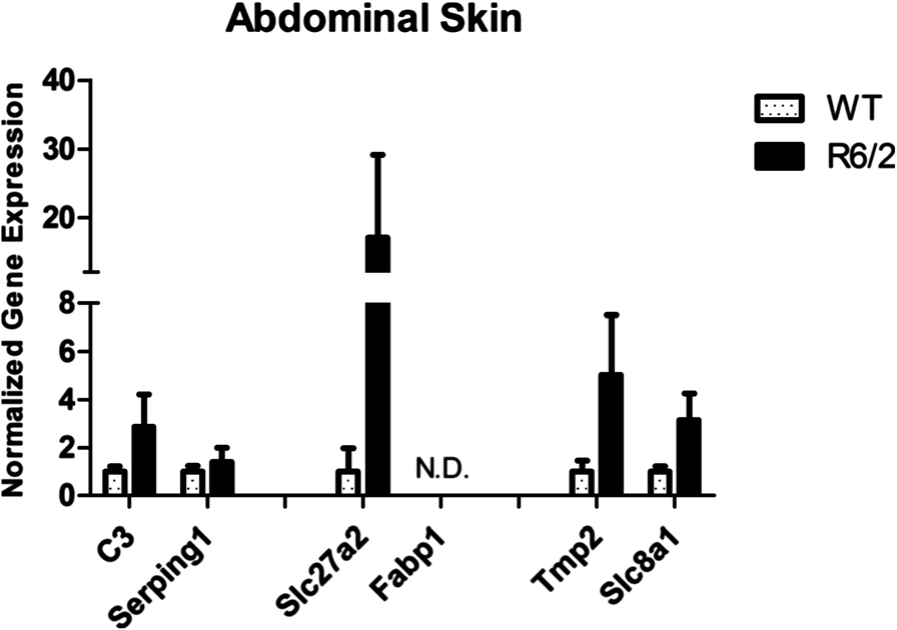
**RT-qPCR in skin.** Expression levels of selected genes were tested in abdominal skin of mice: differences between R6/2 and WT are not statistically significant. *Slc27a2* was measurable in all R6/2 samples and only in one WT mouse; in 2 WT mice where expression was too low to be quantifiable, the values were replaced with detection limit of the qPCR machine: the levels in R6/2 resulted in strong up-regulation with respect to WT. *Fabp1* was not detectable (N.D.).

**Figure 8:**
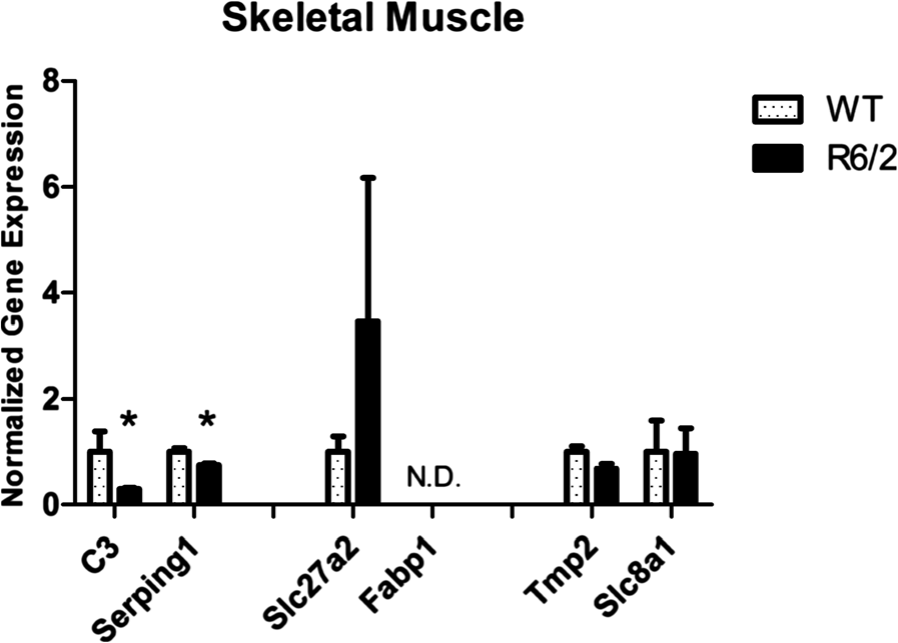
**RT-qPCR in skeletal muscle.** Expression levels of selected genes were tested in skeletal muscle: only *C3* and *Serping1* showed significant down-regulation. *Fabp1* was not detectable (N.D.) (*Student’s t-test p < 0.05 on log expressions).

All genes were tested by RT-qPCR on the same blood samples used for microarray assay and data were normalized on four reference genes. Normalized expression levels confirmed the modulation observed in microarray (reported in Table 2); in detail, *C3*, *Serping1*, *Slc27a2*, and *Fabp1* resulted up-regulated in R6/2 mutant vs WT mice, while *Tpm2* and *Slc8a1* showed down-regulation, although the last one had a small modulation (Figure 6). These results validated peripheral pathway alterations obtained by GSEA, mirroring the fold changes of microarray analysis. Moreover, our data agreed with the dysregulation of complement components that was previously observed in HD patients plasma [23]. On the contrary, in a recent work, *C3* plasma protein level in R6/2 was found not significantly modulated [49] probably due to the different age of animals in our study with respect to theirs. To validate other candidate matrices often employed as source of biomarkers, and to compare peripheral transcriptional alterations, expression levels of the selected genes were investigated in other peripheral tissues, skeletal muscle and skin, where the presence of mut-HTT and polyQ inclusions has been demonstrated [17]. Gene expression alteration has been already investigated in HD skeletal muscle [50-52], but not yet in HD skin, although this tissue has been utilized in transcriptional studies [53]. In our study, *C3* and *Serping1* presented the same modulations in blood and in skin samples but these were not statistically significant (Figure 7); on the contrary, statistically significant down-regulation of these two genes was highlighted in R6/2 skeletal muscle with respect to wild type (Figure 8). *Slc27a2* was not expressed in skin of wild type mice but it was quantifiable only in R6/2 (Figure 7); average up-regulation was also noticed on skeletal muscle samples but with high variability (Figure 8). *Slc27a2* over-expression occurring only in mutant mice allowed us to hypothesize a probable perturbation of fatty acid metabolism in periphery. *Fabp1* was not quantified in these peripheral tissues. *Tpm2* and *Slc8a1* were not significantly modulated in skeletal muscle or skin samples (Figure 7 and 8). Nevertheless, our data partially confirmed a transcriptional modulation for *Tpm2*, which in a previously published work [52] was included in the top 75 decreasing genes in R6/2 quadriceps muscle. Therefore, skeletal muscle may represent a suitable source to investigate peripheral transcriptional alterations; instead, the provided data were not robust enough to validate skin for this purpose. Moreover, this finding suggested that complement pathway is impaired also in skeletal muscle, and the subsequent immune response activation may contribute to muscular atrophy, together with the previously described molecular mechanisms of apoptosis and autophagy [50,52].

**Figure 9:**
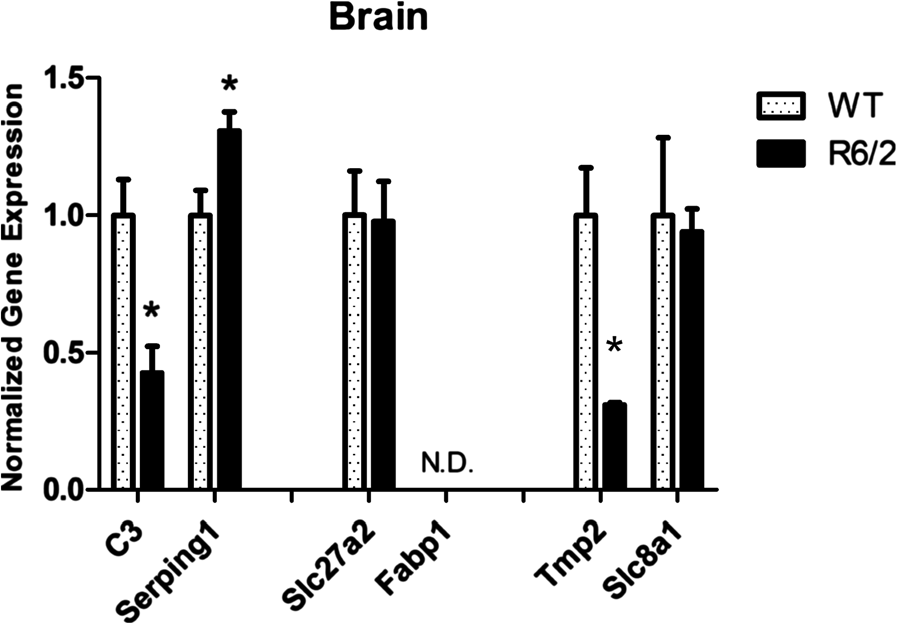
**RT-qPCR in brain.** Normalized expression levels of selected genes showed that *C3* and *Serping1* were modulated but in opposite direction, and *Tmp2* was down-regulated. *Fabp1* was not detectable (N.D.) (*Student’s t-test p < 0.05 on log expressions).

In addition, with the aim to find potential links between blood modulated pathways and the disease etiology and the molecular pathological relationship between peripheral and central tissues, gene expression levels were also analyzed in mouse brains (Figure 9). This investigation revealed that only *C3*, *Tpm2* and *Serping1* were significantly modulated in brain of R6/2, even if the last one highlighted an opposite behavior with respect to the one observed in blood; *Fabp1* resulted undetectable in brain. Up-regulation of other serpins was already observed in R6/2 brain and other tissues as well as in patients brain [54]. In addition, *C3* mRNA increment in R6/2 brains mirrored results of transcriptional investigations displaying an increased complement biosynthesis in different brain areas of HD patients compared to healthy individuals [28,55] but again differed from Larkin et al. [49] that did not observe significant *C3* expression modulation in R6/2 brain. Our data raise the possibility of therapies targeting complement components in neurodegenerative disorder, with probably *Serping1* representing, with respect to *C3*, a better candidate disease target in terms of drug development, and a potential biomarker [30,31].

In conclusion, all these findings enforce the hypothesis that immunological alteration may contribute to neurological degeneration and components of this pathway may represent potential biomarkers to monitor in parallel drug efficacy in periphery and in brain, supporting the usefulness of this model in pre-clinical studies.

## Conclusion

HD is a neurological and devastating disorder with an urgent need of effective therapies and well established biomarkers. Even though major efforts in finding peripheral disease progression biomarkers are pursued in human studies, the availability of validated animal disease models is of great aid in all pre-clinical studies aimed at moving ahead promising compounds into clinical studies, evaluating their possible molecular mechanism of action. In addition, transcriptional analysis on these models could unveil novel therapeutic targets related to the pathology onset and progression.

On the other side, one of the major drawbacks for *in-vivo* pathology models is the incomplete correspondence of these with the real human disease situation that could cause misinterpretation of data, with deleterious effects on drug discovery progression. Here, we evaluated through a whole genome approach, peripheral transcriptional alterations of biological pathways in circulating blood cells of a well characterized HD mouse model. Our data revealed the direct involvement of processes that are well known markers of Huntington’s disease progression in man. Several microarray findings have been investigated by RT-qPCR in other peripheral easily accessible tissues and in brain with obvious projection toward clinical studies. The approach adopted in this work offers interesting remarks that could be further investigated in a larger cohort of transgenic mice at different stages of pathology development and validated in other HD models to strengthen the findings on the involvement in HD progression of these biological pathways obtained by GSEA analysis. Nevertheless, preliminary results presented here could be considered a further step in validating the R6/2 mouse model as first line pre-clinical tool, giving at the same time an insight of important pathological hallmarks for Huntington’s disease, and contributing to attractive novel biomarkers development.

## Methods

### Mouse handling, blood and tissues collection

This study included four R6/2 male mice (about 110 CAG repeats) and four wild type (WT) male littermates that were bred at PsychoGenics Laboratories (Tarrytown, NY, USA). All animals were examined, manipulated, and weighed prior initiation of the study to assure adequate health, suitability and to minimize non-specific stress associated with manipulation. During the course of the study animals were maintained in a 12/12 light/dark cycles with temperature ranging between 20 and 23°C and relative humidity around 50%. Chow and water were provided *ad libitum* for the duration of the study. Genotype was determined by PCR analysis of DNA tail, performed at PsychoGenics Laboratories.

Mice were sacrificed at the age of 16 weeks. Terminal whole blood samples were collected via closed cardiac puncture on anesthetized live mouse (CO^2^). Blood samples were drawn by sterile syringe and 100 μl were directly transferred to RNAprotect Animal Blood Tubes (BD QIAGEN cat n° 76544) according to the manufacturer indications and maintained at −80°C. In addition, the following organs were collected: brain with cerebellum, skeletal muscle (quadriceps) and skin (a strip of about 1 cm × 3 cm of the abdominal area).

All experiments on mice were carried out in strict accordance with the recommendations of the Guide for the Care and Use of Laboratory Animals. The protocol was approved by the Institutional Animal Care and Use Committee of PsychoGenics, Inc. (PHS OLAW animal welfare assurance number A4471-01), an AAALAC International accredited institution (Unit #001213).

### RNA purification

Total RNA was purified from whole blood by RNeasy Protect animal blood kit (QIAGEN GmbH, Hilden, Germany) according to the instruction provided with the kit. Purified RNA was quantified by Nanodrop ND-1000 Spectrophotometer and checked for RNA integrity by Bioanalyzer (Agilent Technologies, Palo Alto, CA). All samples with RIN between 8.40 and 9.0 were included in the subsequent investigations: RNA obtained from blood of a wild type mouse did not satisfy quality control and was excluded from transcriptional investigations (RIN = 7.50).

Total RNA was extracted from brain (ensuring to dissect exactly the same portion, including striatum and cortex), skeletal muscle, and skin after shaving using RNeasy Lipid Tissue Mini kit (QIAGEN) following the instruction provided by the kit.

To assess RNA integrity (28S and 18S ribosomal RNA bands) all samples were loaded on agarose gel with Ethidium Bromide (Sigma-Aldrich Corporation, MO, USA) and quantified by NANODROP 2000 spectrophotometer (Thermo Fischer Scientific, MA, USA).

### Gene expression analysis

#### Microarray

Microarray experiment was performed on individual blood samples, 500 ng of total RNA of each sample was employed to generate target RNA or amplified RNA (aRNA). This aRNA was then hybridized onto GeneChip® Mouse Genome 430 2.0. This array comprises over 45,000 probe sets representing more than 34,000 well-substantiated mouse genes. Microarrays experiment was performed by Precision Biomarker **Resources**, Inc. (Evanston, IL, USA).

#### RT-qPCR

Before cDNA synthesis, the same quantity of RNAs derived from every blood sample of each strain was pooled together obtaining a single pooled sample of wild type mice blood and a single pooled sample of mutant mice blood. The two deriving pools were quantified by NANODROP 2000 Spectrophotometer (Thermo Fischer Scientific, MA, USA). cDNA was synthesized from 1 μg of RNA obtained from blood pooled samples as well as from all the other individual tissues (brain, skeletal muscle and skin), by using QuantiTect Reverse Transcription kit (QIAGEN GmbH, Hilden, Germany) and diluted to 100 μL.

RT-qPCRs on cDNA samples were performed using a CFX96 Real Time System/C1000 Thermal cycler (Bio-Rad Hercules, CA, USA) with iQ™ SYBR Green master mix 2X (Bio-Rad) with primers that specifically amplified target or control genes. The run protocol used in RT-qPCR was the “2 step amplification”, with annealing temperature tailored on the primer pair used. Each run was followed by Melting Curve run and analysis to confirm amplification of a single product.

The primers were designed using Beacon Designer 7.9 software (PREMIER Biosoft International, CA, USA), employing sequences data from NCBI database or from published validated sequences. To guarantee primer specificity, they were checked with NCBI BLAST against Reference RNA sequence (Refseq_rna) of *Mus musculus*. Primers were defined inside the region targeted by Affymetrix probe sets. Complete primer sequences, gene bank accession numbers are shown in Table 3. RT-qPCR expression data on blood samples were normalized on geometric mean of *Ppib*, *Actb*, *Ywhaz* and *Rpl13a*, as described elsewhere [16]. Moreover, *Actb*, *B2m*, *Hprt*, and *Rpl13a* were employed for the other tissues, except *Actb* in skeletal muscle (due to low stability, data not shown). Reference gene primer sequences were taken from Diamanti et al. [16].

**Table 3:** Complete primer sequences and gene bank accession numbers of selected genes analyzed in RT-qPCR

**Gene symbol**	**Ref seq**	**Primer sequence Forward**	**Primer sequence Reverse**
**C3**	K02782, NM_009778	GGTGTGCTGAAGAGAACT	TGAGCCTGACTTGATGAC
**Serping1**	NM_009776	AAAGTAAAGAGCAGCCAAGACA	CAGGTTGAGATCGTAAGTGAAGT
**Slc27a2**	NM_011978	TGAAGAAGTGAATGTGTATGG	TCTCAATGGTATCTTGTATCCT
**Fabp1**	NM_017399	CGTGACTGAACTCAATGG	TTCTCTTGCTGACTCTCTT
**Slc8a1**	NM_011406	TCCATCCAGTAGACTTCGTGAT	CCAAGCAATTCCTTACAGAGTGA
**Tmp2**	NM_009416	AGCCCAAGCGGACAAGTA	CGGGTCTCAGCCTCTTTCA

### Bioinformatics and statistical analyses

Affymetrix CEL files were analyzed with R program (The R Project for Statistical Computing http://www.r-project.org/) by making use of Bioconductor packages (http://www.bioconductor.org). Normalization for quantitative comparison of different microarrays was performed with quantiles within the RMA package [56-58] and final expression values were calculated by summarizing probe set intensities with median-Polish algorithm. Differential expression analysis between four mutant and three wild type samples was tested with LIMMA package [59]. Correction for multiple testing was not performed due to the low number of arrays.

In RT-qPCR, relative expressions, obtained from Cq corrected by amplification efficiency, were normalized on geometric mean of selected reference gene expression values [16].

Differentially modulated genes in brain, skin and muscle among mutant and wild type samples were identified with Student’s t-test on logarithmic transformed expressions, p-values below 0.05 were considered statistically significant. No statistical test was applied on blood samples, because these samples were pooled in RT-qPCR investigations. Statistical analysis of RT-qPCR data was performed in GraphPad Prism 5.

Gene set enrichment analysis of microarray data was performed using GSEA 2.07 software ( [60], http://www.broadinstitute.org/gsea/index.jsp) and using MSigDB v 3.1, focusing attention on the following collections: C2 BioCarta gene sets, C2 KEGG gene sets, C2 Reactome gene sets and C5 GO Biological Processes. Software default parameters were used, except for permutation type parameter that was set to “permutation on gene set” due to the low number of arrays employed. Gene sets with false discovery rate (FDR) less than 0.25 were considered significant.

KEGG pathways were downloaded and processed in R/Bioconductor by using Pathview package [61].

## Competing interests

The authors declare that they have not competing interests.

## Authors’ contributions

DD, DI, CF carried out the transcriptional studies. FM performed functional genomics analysis on probe sets and participated in primer design. EM applied GSEA methods on microarrays data and performed qPCR statistical analysis. LM performed the microarrays statistical analysis. DD and GP conceived the study, and participated in its design and coordination. All authors contributed in preparing the draft manuscript and approved the final version.

## Supplementary Material

Additional file 1 **Enriched GO Biological Process collection.** Description: Table showed Biological Processes enriched in R6/2 (in red) or in WT (in green) and GSEA results. Click here for file

Additional file 2 **Complement and Coagulation Cascades Pathway members part of the core enrichment.** Description: Annotations of genes with corresponding Affy Id are reported together with GSEA statistic (enrichment score, ES) and LIMMA statistics (fold change, FC, and p-value. Click here for file

Additional file 3 **PPAR Signaling Pathway members part of the core enrichment.** Description: Annotations of genes with corresponding Affy Id are reported for PPAR signaling pathway; GSEA statistic (enrichment score, ES) and LIMMA statistics (fold change, FC, and p-value) are also showed. Click here for file

Additional file 4 **Cardiac Muscle Contraction Pathway members part of the core enrichment.** Description: Annotations of genes with corresponding Affy Id are reported together with GSEA statistic (enrichment score, ES) and LIMMA statistics (fold change, FC, and p-value) for Cardiac Muscle Contraction pathway. Click here for file

Additional file 5 **Dilated Cardiomyopathy Pathway members part of the core enrichment.** Description: Annotations of genes with corresponding Affy Id are reported together with GSEA statistic (enrichment score, ES) and LIMMA statistics (fold change, FC, and p-value) for Dilated Cardiomyopathy pathway. Click here for file
